# Energy expenditure and physical activity responses to football for health training in adults with metabolic syndrome: a randomized clinical trial

**DOI:** 10.5114/biolsport.2026.154146

**Published:** 2025-10-01

**Authors:** Athanasios Poulios, Lambros Tsiokanos, Dimitrios Draganidis, Konstantinos Papanikolaou, Panagiotis Tsimeas, Niki Syrou, Georgios Metsios, Athanasios Chatzinikolaou, Georgios Ermidis, Athanasios Tsiokanos, Aggelos Pappas, Magni Mohr, Peter Krustrup, Athanasios Z. Jamurtas, Ioannis G. Fatouros

**Affiliations:** 1 Department of Physical Education and Sport Science, University of Thessaly, Karies, Trikala, Greece; 2 Division of Gerontology, Geriatrics and Palliative Care, Department of Medicine, Heersink School of Medicine, University of Alabama at Birmingham, Birmingham, Alabama, USA; 3 Department of Nutrition and Dietetics, University of Thessaly, Trikala, Greece; 4 Department of Physical Education and Sport Science, Democritus University of Thrace, Komotini, Greece; 5 Department of Sports Science and Clinical Biomechanics, SDU Sport and Health Sciences Cluster (SHSC), University of Southern Denmark, Odense, Denmark; 6 Faculty of Health Sciences, University of the Faroe Islands, Torshavn, Faroe Islands; 7 Danish Institute for Advanced Study (DIAS), University of Southern Denmark, Odense, Denmark; 8 Sport and Health Sciences, University of Exeter, Exeter, United Kingdom

**Keywords:** Recreational football, Caloric expenditure, Blood lactate, Obesity, Dyslipidemia

## Abstract

This investigation determined the energy expenditure (EE), physical activity (PA), and physiological responses of football for health training (FFH). Twenty middle-aged males with metabolic syndrome (MetS) completed a 60-min FFH and a control trial using a randomized crossover design. The FFH load and EE were determined using a mobile gas analyzer, heart rate (HR) monitors, blood lactate measurements, a global positioning system, and accelerometry. Participants in FFH run a total distance of ~3.800 m (1,121 m at > 7 km/h, accelerations of 141 m, decelerations of 162 m) using a mean and maximal speed of 4.1 km/h and 20.6 km/h, respectively. FFH demonstrated a moderate-to-vigorous PA of > 41 min and a step count of ~4900. FFH increased (p < .001) the perceived exertion (55.8%, 13.6 ± 2.6), HR_mean_ (151.1 ± 15.2 beats/min, 83.1 ± 10.9 %HRmax), lactate (80.8%, 5.4 ± 0.9 mmol/L), ${\dot{\text{V}}\text{O}}_{2}$ (88.6%, 1.9 ± 0.3 L/min, 79.9 ± 10.5% ${\dot{\text{V}}\text{O}}_{2\max}$, 6.7 ± 0.8 METs), breathing frequency (32.6 ± 3.0 breaths/min), and respiratory exchange ratio (0.98 ± 0.03) compared to the control trial. Total EE reached 524.2 ± 81.0 kcals (mitochondrial energy production: 476.8 kcals; anaerobic energy production: AS 5.4 ± 1.0 kcals; EPOC: 42.0 ± 11.8 kcals). The present results suggest that FFH meets the international PA and EE standards for adults with MetS, with participants taking part in moderate-to-vigorous PA (MVPA) for approximately 68% of each session, rendering it a suitable and efficient strategy to lower cardiometabolic risk.

## INTRODUCTION

Non-communicable diseases (NCDs) such as cardiovascular diseases, metabolic syndrome, diabetes, cancer etc. are closely linked to a sedentary lifestyle and inadequate levels of physical activity (PA) [1]. The metabolic syndrome (MetS) is a set of interconnected metabolic conditions [i.e. obesity, elevated fasting blood glucose, waist circumference, blood triglycerides, blood pressure and decreased high-density lipoprotein cholesterol (HDL-C)] that predispose individuals to cardiovascular diseases and type 2 diabetes [2]. It is estimated that over a billion people worldwide are affected by MetS [3]. According to the World Health Organization (WHO), obesity and insufficient PA result in over two million deaths/year [4]. Health authorities such as the WHO and the American College of Sports Medicine (ACSM) recommend that adults aged 18–65 should perform ≥ 150 min of moderate-intensity PA, or 75–150 min of vigorous-intensity PA per week, respectively [5, 6]. Furthermore, the accumulation of > 6,000 steps/day is associated with lower odds of developing MetS [7] whereas a caloric expenditure of ~1000 kcal/week reduces the risk of NCDs by ~50% [8].

Systematic exercise is crucial for the prevention and management of MetS [5]. Currently, continuous endurance training (CET) of moderate intensity is primarily recommended for treating MetS [5]. Despite its effectiveness to improve cardiometabolic health and cardiorespiratory fitness in individuals with MetS, CET may be inadequate to enhance musculoskeletal fitness [5]. Resistance training (RT) may not only increase muscle mass and strength, but it can also mitigate MetS-associated conditions [9]. However, combined training (CT) using RT and CET in the same exercise session is mainly adapted as the most effective strategy for MetS to improve overall cardiometabolic health and fitness [9]. Although CT may be effective, it is characterized by low adherence due to its large time investment (300–400 min/week) and is less motivating [10]. However, interval-type training (INT), which introduces mainly cardiovascular activities using periods of alternating lower and higher intensity, is a promising and time-efficient mode towards the improvement of cardiorespiratory fitness and management of MetS [9]. Finally, hybrid-type training has the characteristics of INT, but it engages both the cardiovascular and musculoskeletal systems during the same exercise session and thus inducing both cardiovascular- and musculoskeletal-type adaptations that could aid the management of Mets [9, 11]. Hybrid-type interventions are time-efficient and entertaining, resulting in low attrition rates [9]. Moreover, a recent systematic review summarized evidence from controlled trials and reported that recreational team sports (e.g., football, basketball) lead to significant improvements in BMI, systolic blood pressure, cholesterol, triglyceride levels, and aerobic fitness in adults with overweight and obesity populations compared to inactive controls [12].

Football for Health training (FFH) clearly has the characteristics of hybrid-type training, engaging both the musculoskeletal and cardiorespiratory systems [13]. FFH, which is characterized by modification in terms of the player’s number, distances, durations, and rules [14], contributes positively to various conditions such as MetS, diabetes, hypertension, obesity, and cancer [15, 16]. Moreover, 45–60 minutes of FFH for 12–16 weeks upgrades ${\dot{\text{V}}\text{O}}_{2\max}$ by 5–15% and muscle power by 6.8% [15, 17]. Additionally, FFH results in favorable changes in fat mass, muscle mass, bone mineral content and mitochondrial function in adults while increasing motivation and adherence to exercise [18–20].

Although FFH has become a popular exercise modality among individuals with NCDs [18], its energy expenditure has not been investigated for adults with MetS. Previous research indicates that FFH induces an average heart rate (HR_mean_) of ~80–90% of maximum (%HRmax) [21] that corresponds to an intensity of ~8 METs [22]. Without assessing the post-exercise energy expenditure, it has been estimated that 1-h of 5-a-side futsal practice induces an energy cost of ~634 kcal [22]. However, there is a methodological limitation in the determination of total energy expenditure (TEE) because football is an intermittent activity with frequent activity changes and a high number of powerful actions [23]. As a result, both mitochondrial and non-mitochondrial energy sources (phosphagens, lactic acid system) are heavily utilized during football practices and games, which may contribute to the rise of oxygen consumption following exercise (EPOC) as compared with cardiovascular-type exercises during which ${\dot{\text{V}}\text{O}}_{2}$ may reach a steady state [24].

Therefore, this investigation aimed to determine (i) the physiological responses and TEE associated with FFH in middle-aged males with MetS and (ii) whether FFH meets the international PA standards set by WHO and ACSM for middle-aged men. The control group was designed to represent typical sedentary behavior among adults with metabolic syndrome, helping to isolate the effects of the FFH intervention relative to typical lifestyle behavior [25]. Our null hypothesis was that FFH (i) will not induce a clinically meaningful TEE; and (ii) will not meet the guidelines for PA set by the WHO and ACSM.

## MATERIALS AND METHODS

### Participants

**FIG. 1:**
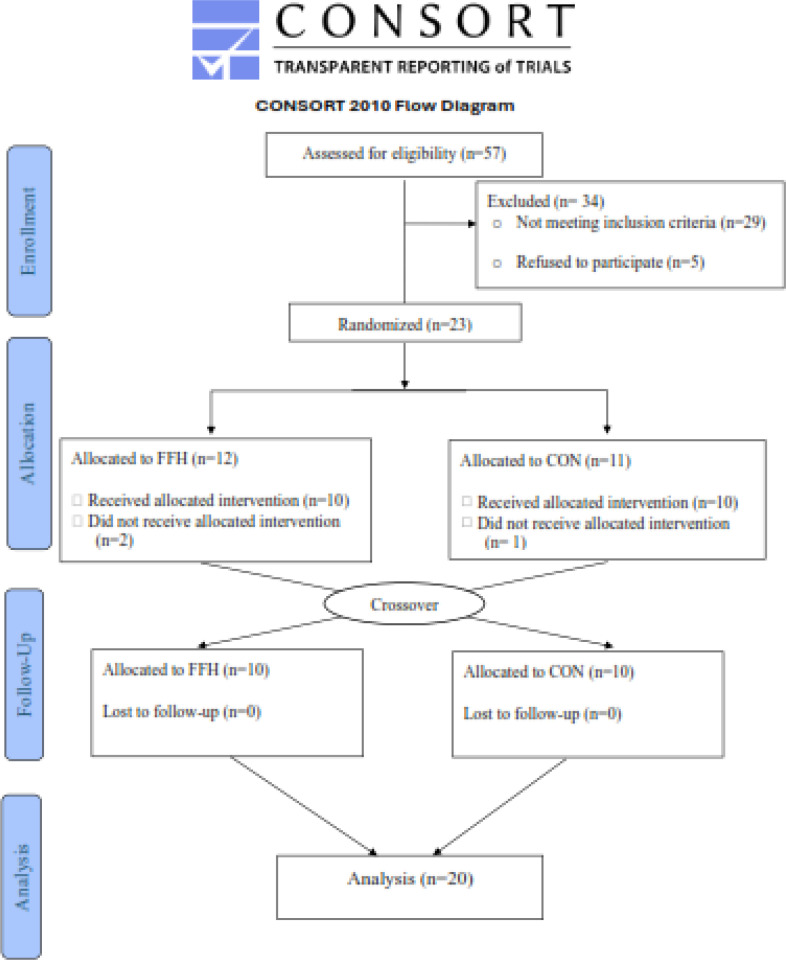
The CONSORT 2010 flow diagram of the study. FFH, football for health training trial; CON, control trial.

A power analysis indicated that ≥ 10 participants would be an appropriate sample size (with a power of 0.90, an effect size of 0.25, and a probability error of 0.05) for determining statistical significance. Initially, 57 participants were interviewed; 23 of them were included in the study, and data from 20 individuals was analyzed (Figure 1). Volunteers were recruited by word of mouth and fliers. Participation was secured if participants: a) were 40–65 years old; b) met the clinical criteria for MetS (≥ 3 of five risk factors) excluding diagnosed diabetes [2]; c) had not consumed alcohol, supplements or tobacco for > 6 months before the study; d) were sedentary (< 7500 steps/ day) and had a low cardiorespiratory fitness level (${\dot{\text{V}}\text{O}}_{2\max}$ should be below the 30^th^ percentile of the corresponding sex and age of the ACSM norms) [6]; e) had not had a weight loss > 10% of their body mass in the last 6 months before the study; f) were free of cardiovascular events, psychiatric disorders, and musculoskeletal limitations. Table 1 presents participants’ characteristics. Following an analytical explanation of the study’s goals and procedures, the participants signed a written consent form and answered a series of medical questions. The Helsinki Declaration’s (https://sites.jamanetwork.com/research-ethics/index.html) standards were followed in the conduct of this study, which received Institutional Ethics Committee approval (No. 2374). This study was registered at ClinicalTrials.gov (NCT06477705).

**TABLE 1:** Participants’ characteristics (N=20)

Age (years)	49.3 ± 5.0
Body mass (kg)	87.2 ± 9.1
Height (m)	1.75 ± 0.04
BMI (kg/m^2^)	28.5 ± 2.4
Body fat (%)	33.0 ± 3.3
Fat Mass (kg)	28.9 ± 5.0
Waist circumference (cm)	105.0 ± 3.7
HR_rest_ (beats/min)	75.2 ± 3.9
HR_max_ (beats/min)	171.2 ± 5.0
${\dot{\text{V}}\text{O}}_{2\max}$ (ml/min/kg)	29.8±4.4
Systolic (mmHg)	133.1 ± 3.9
Diastolic (mmHg)	84.4 ± 3.5
Triglycerides (mg/dL)	174.7 ± 6.8
HDL cholesterol (mg/dL)	28.8 ± 4.0
Fasting glucose (mg/dL)	104.1 ± 4.2
RMR (kcal/day)	1,693 ± 249
Habitual physical activity (steps/day)	5,444 ± 1,535

BMI, body mass index; HRrest, Heart Rate rest; HRmax, Heart Rate max; ${\dot{\text{V}}\text{O}}_{2\max}$, maximal oxygen consumption; HR_rest_, resting heart rate; HR_max_, maximal heart rate; HDL, High Density Lipoprotein; RMR Resting Metabolic Rate.

### Experimental design

**FIG. 2:**
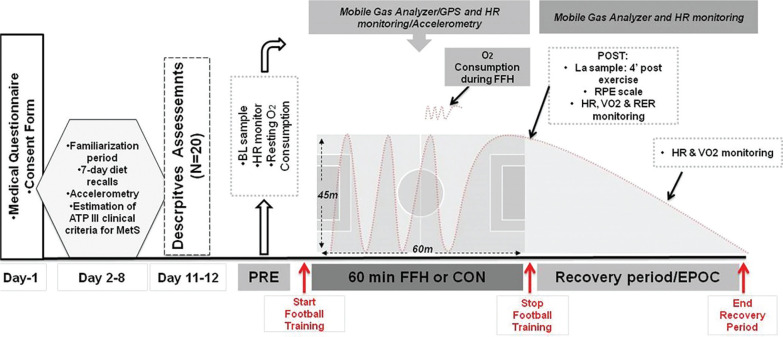
The experimental flowchart. MetS, metabolic syndrome; FFH, football for health training; CON, control trial; N, number of participants; La, blood lactate; HR, heart rate; ${\dot{\text{V}}\text{O}}_{2}$, oxygen consumption; GPS, global positioning system; RPE, rating of perceived exertion; RER, respiratory exchange ratio; EPOC, post exercise oxygen consumption.

A two-trial [FFH vs. control trial (CON)], randomized, cross-over, repeated measures design was applied (Figure 2). Participants had their body mass, height, waist circumference, and blood pressure measured and provided a blood sample to measure the concentration of triglycerides, HDL-cholesterol, and glucose to verify the occurrence of MetS. A certified dietitian instructed the participants on how to record 7-day diet recalls and guided them to adjust to a weight-maintenance diet during a 1-week familiarization phase with the experimental procedures. Thereafter, participants were requested to maintain the same dietary plan during the study period. During familiarization, participants’ daily habitual PA was assessed using accelerometry as previously described [26]. After the familiarization period, participants were subjected to baseline measurements [body composition, resting metabolic rate (RMR), resting heart rate (HR_rest_), resting blood pressure (BP) and ${\dot{\text{V}}\text{O}}_{2\max}$]. Finally, participants were randomized using a balanced algorithm into two trial conditions: FFH and CON, with a crossover after a washout period. Each particpant completed both trials in a randomiazed order. Before each trial (PRE), the rating of perceived exertion (RPE), HR, respiratory exchange ratio (RER) and blood lactate (La) were assessed. During FFH and CON, the HR and oxygen consumption (${\dot{\text{V}}\text{O}}_{2}$), were continuously monitored in order to determine the internal load. Field performance and PA levels were also measured during the 60-minute FFH. Immediately after (POST) each trial RPE, RER, ${\dot{\text{V}}\text{O}}_{2}$ and La were assessed again. Then, the HR and ${\dot{\text{V}}\text{O}}_{2}$ were monitored until they returned to baseline levels, determining excess post-exercise oxygen consumption (EPOC).

#### Football for health training

The 60-min [10-min warm-up, 10-min fitness drills, 10-min football technical exercises, 30-min small-sided games (7 v 7, 45 × 60 m)] FFH was performed on an outdoor field with natural grass by a professional football coach at 08:00 p.m. according to guidelines previously published [27]. The participants received technical guidance, comments, and feedback. The participants were only allowed to consume water *ad libitum*. The participants in CON were sitting on a chair for 60 minutes. Following the trials, participants were required to stay seated until the HR, La, and ${\dot{\text{V}}\text{O}}_{2}$ returned to their pre-exercise values, assessing the EPOC via a gas analyzer and HR monitor.

### Assessment of physiological responses, energy expenditure, and field activity during training

The physiological responses were measured using the following metrics: ${\dot{\text{V}}\text{O}}_{2}$ (and as % ${\dot{\text{V}}\text{O}}_{2\max}$), estimated metabolic equivalents (MET’s), La, breathing frequency (BF), RPE, respiratory exchange ratio (RER), and HR_mean_ (and as % HRmax attained during ${\dot{\text{V}}\text{O}}_{2\max}$ evaluation). Field activity (total distance, mean speed, speed zones, accelerations, and decelerations) during training was recorded using a high-resolution global positioning system (Appex Team Series, STAT Sports, UK) as previously described [28]. The number of steps taken and the intensity of PA were recorded via accelerometry (GT3X+, Acti-Graph, Pensacola, FL, USA) as previously described [26]. RPE was measured using the 6–20 Borg scale. HR was monitored using a portable HR monitor (Polar Electro-Oy HR monitor, Kempele, Finland), and mean and maximal HR were recorded. La was assessed at rest and 4-min post-exercise using capillary blood that was measured (on single-use strips) on a hand-held analyzer (Lactate Plus^ΤΜ^, Nova Biomedical, USA) as previously described [29]. Portable indirect calorimetry (Vmax, Sensormedics, Yorba Linda, CA) was utilized to measure the energy cost at rest and during the FFH and recovery (EPOC) as previously described [29].

The following elements [30] were summed for the TEE calculation: a) the calories associated with the mitochondrial energy pathway (MEP) were calculated using the formula [aerobic energy expenditure: (${\dot{\text{V}}\text{O}}_{2\text{exercise}}-{\dot{\text{V}}\text{O}}_{2\text{rest}}$) × 21.1 (kJ) / 4.184]; b) the calories associated with the anaerobic energy system (AS) were calculated using the formula [(La_rest_ – La_post-exercise_ × Body Mass (kg) × 3.0 (ml O_2_)] × 21.1 (kJ) / 4.184]; and c) the EPOC was calculated as [(${\dot{\text{V}}\text{O}}_{2\text{excess}}-{\dot{\text{V}}\text{O}}_{2\text{rest}}$) × 19.6 (kJ) / 4.184] × Duration excess (min).

### Anthropometrics, body composition, and haemodynamic measurements

Body mass and height were evaluated using a beam balance and a stadiometer (Beam Balance-Stadiometer, SECA, Vogel & Halke, Hamburg, Germany) and the body mass index was then computed [31]. The waist circumference was measured using a Gullick II tape. Body composition was estimated using dual-emission X-ray absorptiometry (DXA, GE Healthcare, Lunar DPX NT, Diegem, Belgium) with participants in a supine position as previously described [32]. Systolic (SBP) and diastolic (DBP) blood pressures were measured at 8.00 a.m. after an overnight fast and a 2-h rest using standard procedures (once every 30-min over the 2-h resting period, and the average value was recorded). HR_rest_ was measured during the same time intervals as for BP.

### Performance measurements

The graded exercise testing protocol (GXT), as previously described [33], was used to estimate the ${\dot{\text{V}}\text{O}}_{2\max}$ on a treadmill (Stex 8025 T, Korea) using an open-circuit spirometry breath-by-breath system (Vmax Encore 29, BEBJO 296, Yorba Linda, CA, USA). The resting metabolic rate (RMR) was evaluated using an open-circuit canopy hood module system (Vmax Encore 29, BEBJO 296, Yorba Linda, CA, USA) for 30–40 min under fasting conditions as previously described [28]. HR was recorded continuously during the GXT protocol using a Polar Electro-Oy HR monitor (Keple, Finland).

### Blood sampling and assays

A resting blood sample was obtained into tubes containing ethylenediaminetetraacetic acid under fasting conditions by venipuncture using a disposable needle (20-gauge) and a Vacutainer tube holder from an antecubital arm vein in a sitting position in the morning and left at room temperature for 20-min to clot. Serum was obtained by centrifugation (1370 g, 4°C, 10-min), the supernatant was transferred into Eppendorf tubes and samples were stored at −80°C in multiple aliquots until assayed in duplicate using a clinical chemistry analyzer (Z1145, Zafiropoulos Diagnostica S.A., Greece) and a commercially available kit (Zafiropoulos Diagnostica S.A.) to determine the concentration of fasting glucose, triglycerides, and HDKcholesterol concentration as previously decribed [34].

#### Statistical analysis

Data are presented as mean ± SD. The normality check for all dependent variables was examined via the Shapiro-Wilk test and was found to be significantly different from normal for La, ED, HR, ${\dot{\text{V}}\text{O}}_{2}$, AS, and EPOC values, which were analyzed using the Wilcoxon test and Friedman test for multiple comparisons. The rest of the dependent variables were analyzed using a two-way (trial X time) repeated measures ANOVA. The Bonferroni multiple comparison test was used when a statistically significant effect was found. For the RPE variable, a two-way repeated measure ANOVA with Greenhouse-Geisser correction was selected. Differences between the interventions were estimated via paired t-tests. Effects sizes (ES) and confidence intervals (CI) were estimated for each dependent variable using the corrected for Hedge’s g technique. ES was categorized as none, small, medium-sized, and large, for levels 0.00–0.19, 0.20–0.49, 0.50–0.79, and ≥ 0.8, respectively. Statistical significance was accepted if p < .05.

## RESULTS

Participants did not report any injuries during this study. The study’s sample met (Table 1) the criteria for MetS and was homogeneous in terms of anthropometric characteristics and physical conditioning level. The lack of differences in all dependent variables at baseline in both trials proves that a one-week wash-out between trials was effective in that aspect. FFH was characterized (Table 2) by a total distance of ~3.800 m, a mean speed of 4.1 km/h, a maximal speed of 20.6 km/h, 1,121 m running at speeds > 7 km/h (410 m at speeds > 11 km/h), a total distance of accelerations of 141 m and decelerations of 162 m. In respect to PA (Table 3), FFH was characterized by a moderate-to-vigorous PA of > 41 min (or ~70% of training time) and a total step count of > 4900.

**TABLE 2:** Field Locomotor activity during 60-min FFH (N=20)

Field Markers	Mean ± SD
Total Distance (m)	3778.8 ± 680.1
Distance < 3.00 km/h (m)	414.8 ± 150.5
Distance 3.00–6.99 km/h (m)	2242.8 ± 402.3
Distance 7.00–10.99 km/h (m)	707.4 ± 248.6
Distance 11.00–14.99 km/h (m)	327.6 ± 144.6
Distance 15.00–18.99 km/h (m)	77.3 ± 37.4
Distance > 19 km/h (m)	9.0 ± 7.8
ACC 1.00–1.99 m/s^2^ (No)	122.1 ± 34.8
ACC 2.00–2.99 m/s^2^ (No)	18.1 ± 7.2
ACC > 3.00 m/s^2^ (No)	1.0 ± 0.8
DEC 1.00–1.99 m/s^2^ (No)	141.1 ± 35.2
DEC 1.00–1.99 m/s^2^ (No)	19.8 ± 8.1
DEC > 3.00 m/s^2^ (No)	1.2 ± 0.8
Max Speed (km/h)	20.6 ± 1.0
Average Speed km/h (m)	4.1 ± 0.8
Sprints Number (No)	1.4 ± 1.0
Maximal HR (beats/min)	192 ± 21
Average HR (beats/min)	137 ± 16
% of TD at < 60% of HRmax (%)	16.2 ± 9.6
% of TD in 60–69% of HRmax (%)	13.7 ± 15.0
% of TD in 70–79% of HRmax (%)	15.5 ± 12.4
% of TD in 80–89% of HRmax (%)	27.2 ± 20.5
% of TD > 90% of HRmax (%)	27.5 ± 30.4

FFH, football for health training; ACC, Acceleration; DEC, Deceleration; HR, Heart Rate; TD, Total Distance; Max, Maximum

**TABLE 3:** The daily assessment of quantity and intensity of habitual PA and PA during 60-min Football for Health Training using accelerometry (N=20)

Markers	Habitual Physical Activity (Mean ± SD) (% of total daily PA time)	60-min FFH (Mean ± SD) (% of total FAM time)
Sedentary (min)	1171.0 ± 95.9 (86.1%)	6.6 ± 2.5 (11.0%)
Light (min)	170.2 ± 46.5 (12.4%)	11.3 ± 4.5 (18.8%)
Moderate (min)	14.4 ± 6.1 (1.0%)	36.8 ± 6.3 (61.4%)
Vigorous (min)	4.1 ± 1.0 (0.3%)	4.9 ± 2.8 (8.2%)
Very Vigorous (min)	0.7 ± 0.2 (0.05%)	0.1 ± 0.1 (0.1%)
Total MVPA	18.9 ± 8.1 (1.3%)	42.2 ± 5.5 (69.6%)
Step Counts	5444.0 ± 1534.9	4903.7 ± 850.3

PA, Physical Activity; MVPA, Moderate-to-Vigorous Physical Activity; FFH, football for health training; SD, standard deviation.

### Physiological Responses

**FIG. 3:**
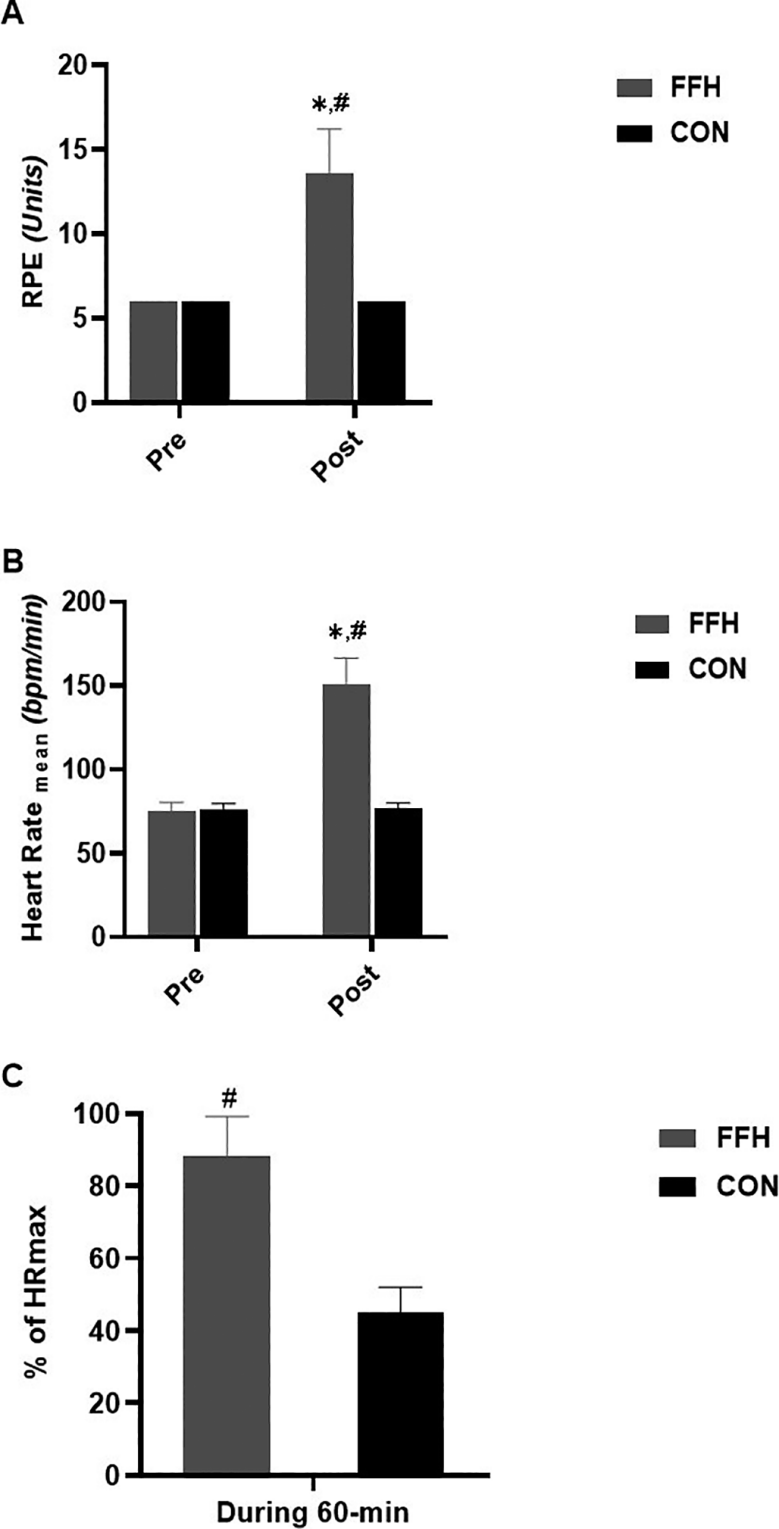
Changes in the rating of perceived exertion (A) and mean (B) and maximal (C) heart rate responses during the control and the FFH. Means and standard deviations are presented via vertical bars (N=20). % of HRmax values presented in Figure 3c represents the mean heart rate expressed as a percentage of the individual’s maximum heart rate; RPE, rating of perceived exertion; HR, heart rate; FFH, football for health training trial; CON, control trial. ^*^denotes differences with Pre; ^#^denotes differences between trials.

RPE (Figure 3A) increased in response to FFH (55.8%, p < .001; 95% CI = -5.10 to -2.94; ES = -4.02) but not in response to CON. The mean (Figure 3B) and mean as a percentage of maximal (Figure 3C) HR increased (HR_mean_: 50.11%, p < .001; 95% CI = -8.09 to -4.97; ES = -6.53; %HRmax: 49.1%, p < .001; 95% CI = 5.02 to 8.17; ES = 6.60) during FFH but not during CON. The HR_mean_ during FFH, as a percentage of HRmax was higher during FFH compared to CON (49.1%, p < .001; 95% CI = 3.88 to 6.46; ES = 5.17).

**FIG. 4:**
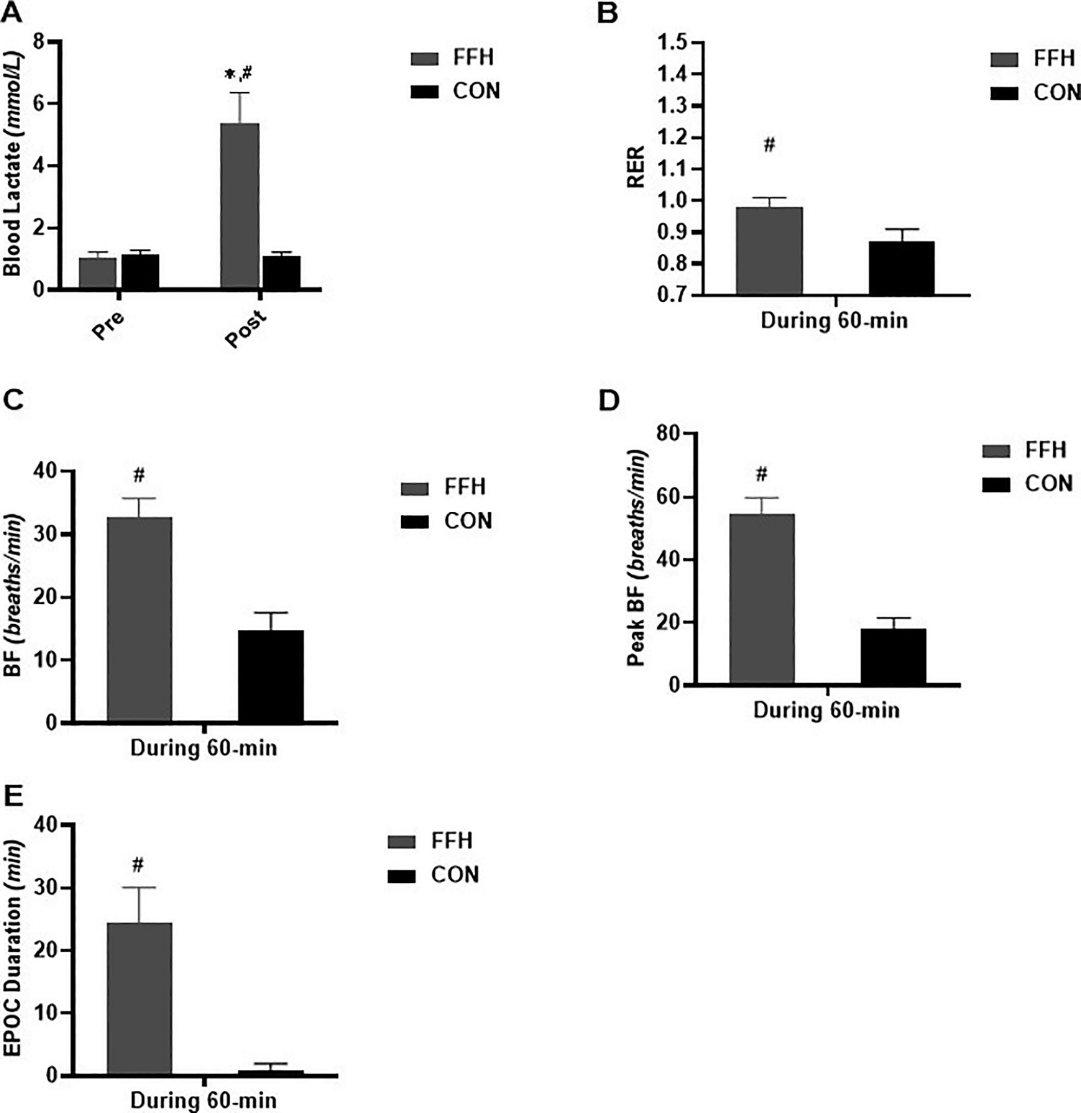
Blood lactate (A), respiratory exchange ratio (B), mean (C) and peak (D) breathing frequency, and ΕPOC duration (E) responses during the control and the FFH. Means and standard deviations are presented via vertical bars (N=20). BL, blood lactate; RER, respiratory exchange ratio; BF, breath frequency; EPOC, post exercise oxygen consumption; FFH, football for health training trial; CON, control trial. ^*^denotes differences with Pre; ^#^denotes differences between trials.

The La concentration in FFH (Figure 4A) increased (80.8%, p < .001; 95% CI = -7.42 to -4.52; ES = -5.97) only in response to FFH. The mean RER (Figure 4B), the mean BF (Figure 4C), peak BF (Figure 4D) and ED (Figure 4E) were higher during FFH compared to CON by 11.2% (p < .001; 95% CI = 2.14–3.96; ES = 3.05), 54.9% (p < .001; 95% CI = 4.44–7.30; ES = 5.87), 66.9% (p < .001; 95% CI = 6.59–10.54; ES = 8.56) and 95.8% (p < .001; 95% CI = 4.23–6.98; ES = 5.61–95%) respectively.

**FIG. 5:**
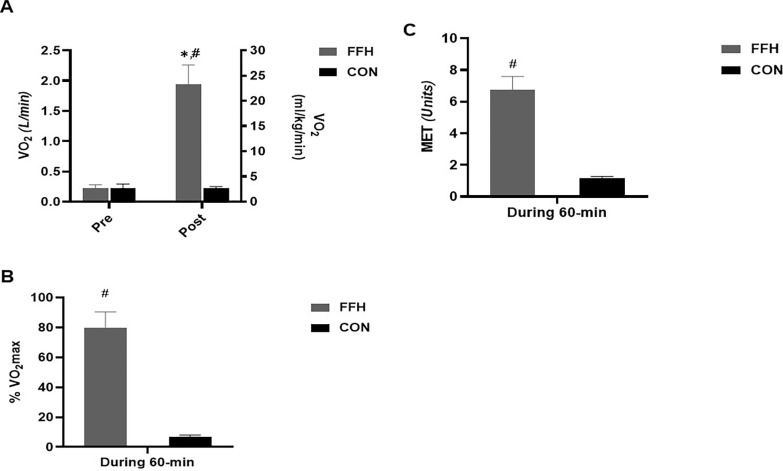
Changes in ${\dot{\text{V}}\text{O}}_{2}$ in absolute and relative values (A), ${\dot{\text{V}}\text{O}}_{2\text{mean}}$ as a percentage of ${\dot{\text{V}}\text{O}}_{2\max}$ (B), and the MET count (C) during the control and the FFH. Means and standard deviations are presented via vertical bars (N=20). ${\dot{\text{V}}\text{O}}_{2}$, oxygen consumption; ${\dot{\text{V}}\text{O}}_{2\max}$, maximal oxygen consumption; MET, metabolic equivalent task; FFH, football for health training trial; CON, control trial. ^*^denotes differences with Pre; ^#^denotes differences between trials.

The ${\dot{\text{V}}\text{O}}_{2}$ (Figure 5A) and the mean ${\dot{\text{V}}\text{O}}_{2}$ (Figure 5B) increased during FFH by 88.6% (p < .001; 95% CI = -9.04 to -5.60; ES = -7.32) and 91.6% (p < .001; 95%CI = 7.35–11.71; ES = 9.53), respectively. No change was observed in CON. The MET units increased only during FFH (Figure 5C) by 82.6% (p < .001; 95% CI = 7.04–11.23; ES = 9.14).

### Energy expenditure

**FIG. 6:**
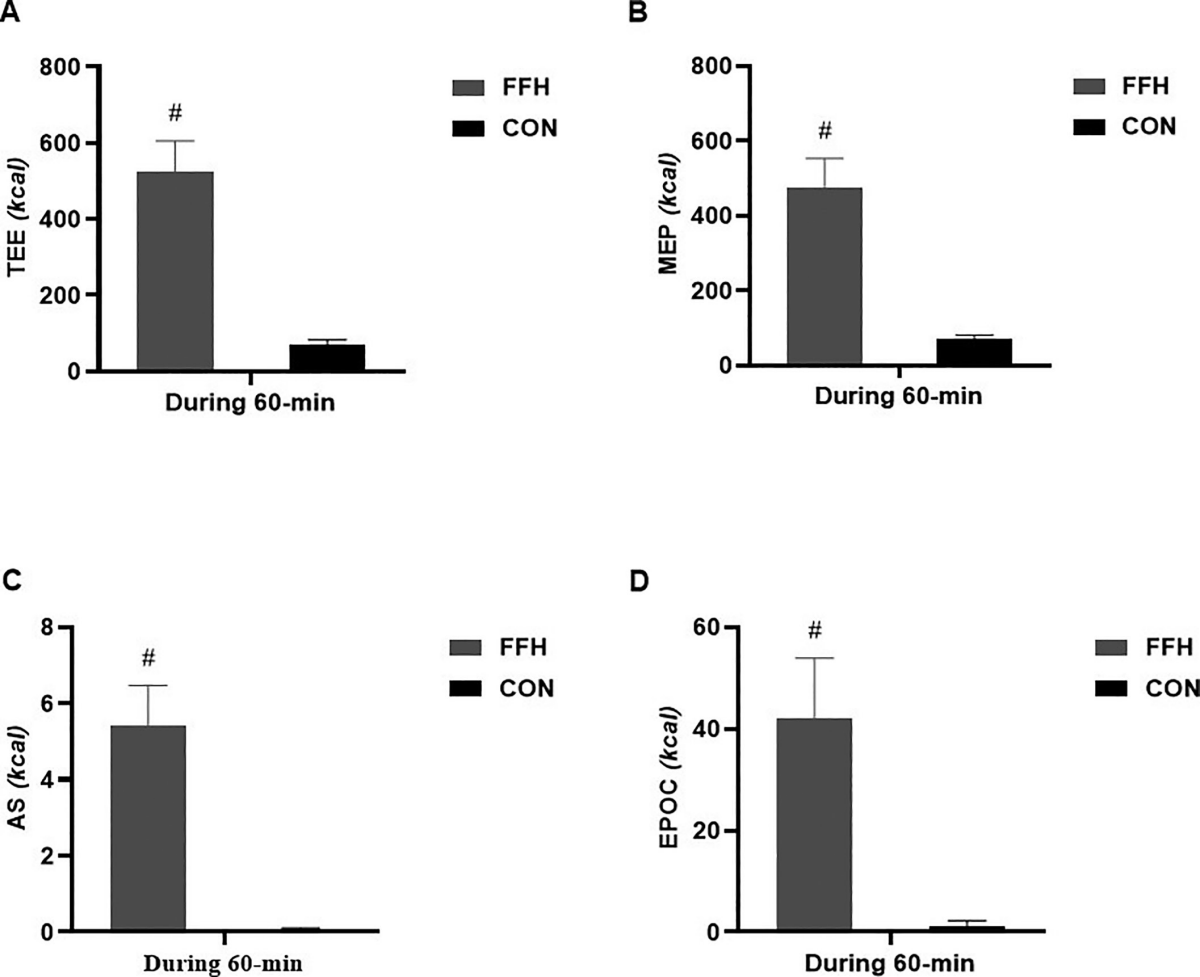
Changes in total energy expenditure (A), energy derived from the mitochondrial energy pathway (B), energy derived from the anaerobic system (C), and energy derived from post-exercise oxygen consumption (D) in the control and the FFH. Means and standard deviations are presented via vertical bars (N=20). TEE, total energy expenditure; MEP, mitochondrial energy pathway; AS, anaerobic system; EPOC, post-exercise oxygen consumption; FFH, football for health training trial; CON, control trial. ^#^denotes differences between trials.

The TEE (Figure 6A) increased in response to FFH (86.5% (p < .001; 95% CI = 5.88–9.46; ES = 7.76) but not in response to CON reaching a value of 524.5 ± 81.1 Kcals or 10.4 ± 2.4 Kcals/min. The components of TEE, i.e. the MEP (Figure 6B), AS (Figure 6C), and EPOC (Figure 6D) increased only in response to FFH (MEP: 85.4%, p < .001; 95% CI = 5.57–9.00; ES = 7.29; 476.8 Kcals or 8.6 ± 1.8 Kcals/min – AS: 99%, p < .001; 95% CI = 5.34–8.65; ES = 7.00; 5.4 ± 1.0 Kcals or 0.1 ± 0.1 Kcals/min – EPOC: 97.2%, p < .001; 95% CI = 2.78–5.33; ES = 4.05; 42.0 ± 11.8 Kcals or 1.7 ± 0.4 Kcals/min).

The MEP-related energy expenditure (Table 4) was higher compared to the AS (FFH: 98.8%, p < .001; 95% CI = 6.56–10.50; ES = 8.53 – C: 99.2%, p < .001; 95% CI = 6.65–10.6; ES = 8.65) and EPOC components (FFH: 91.1%, p < .001; 95% CI = 5.96–9.59; ES = 7.78 – C: 98.3%, p < .001; 95% CI = 5.24–9.20; ES = 7.22) in both trials. The AS-related energy expenditure was lower than the EPOC-related energy expenditure only in FFH (87.1%, p = .033; 95% CI = -5.38 to -3.14; ES = -4.26).

**TABLE 4:** The differences among the MEP, the AS and EPOCassociated energy expenditure in both trials (N=20).

Energy Expenditure System	FFH (Mean ± SD)	CON (Mean ± SD)
MEP (kcals)	476.9 ± 76.6	69.9± 11.2
AS (kcals)	5.41 ± 1.06^1^	0.05 ± 0.06^1^
EPOC (kcals)	42.00 ± 11.86^1^, ^2^	1.14 ± 1.15^1^

MEP, mitochondrial energy pathway; AS, anaerobic system; EPOC, excess post-exercise energy cost; FFH, football for health training trial; CON, control trial;

1 Difference with MEP;

2 Difference with AS; SD, standard deviation.

## DISCUSSION

This study provides evidence that, in adults with MetS, FFH (i) is associated with a TEE of 525 kcal (8.6 Kcal/min) during a 60-min session with the MEP contributing to a greater extent than AS and EPOC; (ii) elicits a substantial physiological metabolic response and (iii) when performed at least twice per week meets the standards for PA and energy expenditure set by international organizations such as the ACSM and the WHO.

The actual physiological response and activity profile of football training depend on variables including intensity, duration, and density [18, 28]. These variables were measured for FFH via field locomotor action using GPS instrumentation, ${\dot{\text{V}}\text{O}}_{2}$-related measures, La, and HR [28, 35, 36]. Previous studies have shown that the markers of internal and external load during small-sided football are reproducible [37]. In this trial, a recreational 60-min FFH was associated with a total distance of ~4,000 m with ~400 m of them performed at a relatively high intensity. Additionally, the average HR and La in this study were measured at 88.3% of HRmax and 5.4 mM, respectively, coinciding with values identified in previous studies for these variables [27, 38]. The mean BF increased by 54.9% with the peak BF reaching 55 breaths/min, suggesting a high-intensity effort. It is characteristic that BF levels during FFH were similar to those seen during cycling HIIT protocols [35]. It has been suggested that the RPE may rise by 78% after a 60-min FFH [38] despite the fact that the RPE elevation was lower (55.8%) in this study. Since there is a strong relationship between RPE, BF and exercise duration [17], and knowing that the FFH duration was similar between studies, an alternative explanation for this discrepancy is the high eccentric component of FFH. In this study participants performed 255 fewer movements with a strong eccentric component (e.g., sprints, accelerations, decelerations, and change of directions) compared to previous studies [38].

**FIG. 7:**
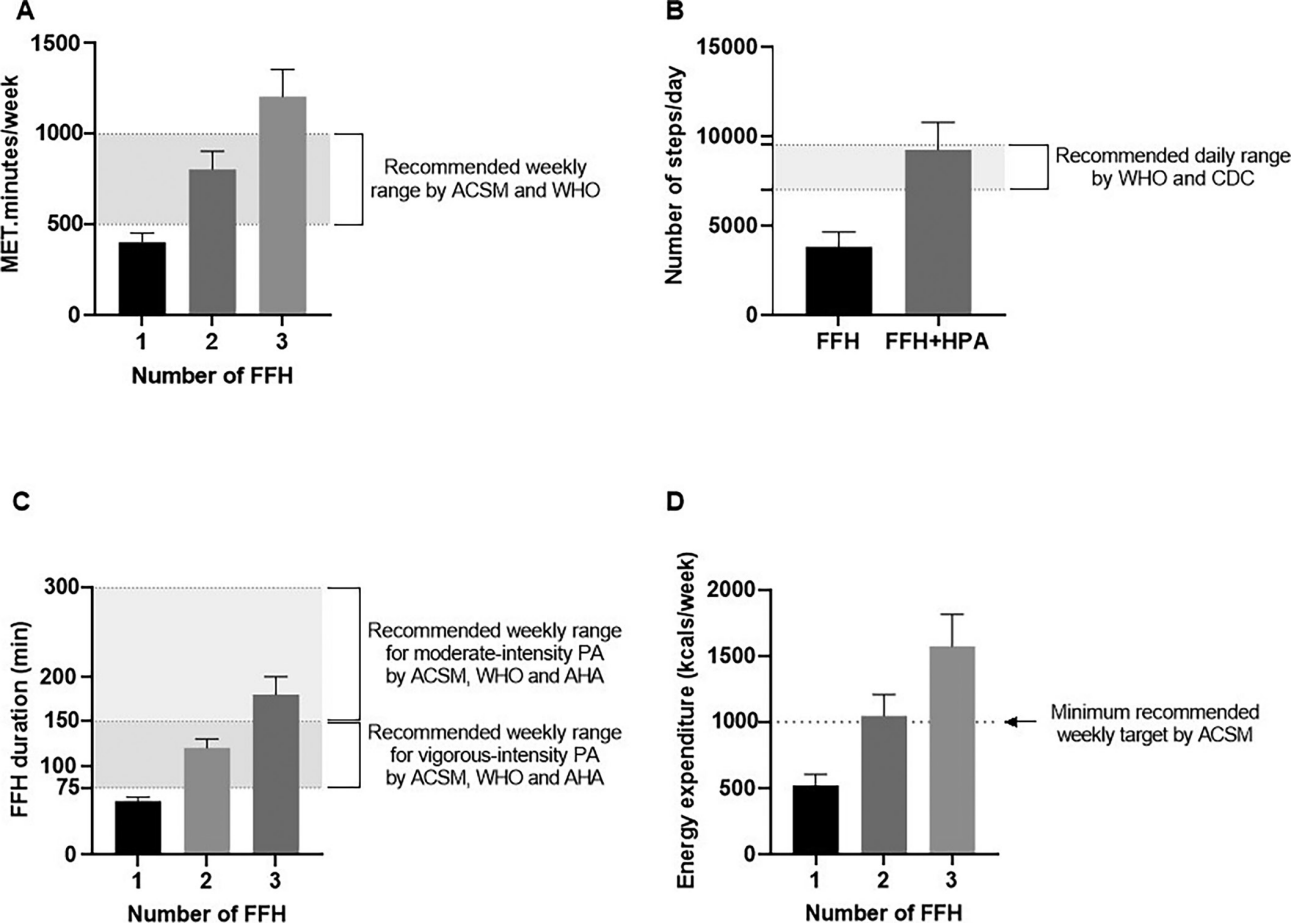
Evaluation of the FFH values according to ASCM, WHO, CDC and AHA recommendations in respect to exercise duration (A), step count (B), energy expenditure (C) and MET count (D). The x axis shows the number of weekly training sessions. The data of the 2^nd^ and 3^rd^ FFH are based on estimations based on a single FFH training session. ACSM, American College of Sports Medicine; WHO, World Health Organization; FFH, football for health training; CDC, Center of Disease Control and Prevention; AHA, American Heart Association; EE, energy expenditure; min; minutes; No, number; MET, Metabolic Equivalent of Task.

As mentioned by the ACSM, the classification of exercise intensity has been determined in the middle-aged group as light when MET level is 2.0–3.9, moderate when the MET level is 4.0–5.9, and vigorous when the MET levels exceed 6.0 [8]. Here, the MET level was estimated at 6.7 units, suggesting that FFH may be categorized as a vigorous exercise protocol for men with MetS, which can develop fitness status or reduce cardiometabolic risk based on ASCM recommendations [6]. Several studies recommend that most adults be encouraged to engage in moderate-intensity cardiorespiratory exercise or combine moderate-to-vigorous exercise to achieve a total of 500–1000 MET · minute/week [34, 39]. This study found that one session of FFH corresponds to 402 MET/minutes, suggesting, using an estimation method that participation in a second or third FFH (figure 7A) on a weekly basis may correspond to ~804–1206 MET · minute/week and such levels have been documented to lower the rates of CVD and premature mortality and enhance cardiorespiratory fitness [40]. A rise of daily steps by > 2,000, or the execution of ≥ 7,000–10,000 steps/day, has been associated with BMI and fat mass reductions [8, 39], a rise of HDL-cholesterol concentration by 19%, and a decline of triglyceride concentration, SBP and fasting glucose by 27.8%, 6%, and 4%, respectively, in adults with MetS [40]. Interestingly, FFH was associated with ~4,900 steps/session, which is 47% higher than the recommended increase of the daily step count [8]. Also, it is noted that the summation of the daily habitual PA step count and the number of steps taken during FFH corresponds to > 10,000 steps (figure 7B). Therefore, FFH could contribute immensely to reaching the daily PA goals, and as such, enhance the health and well-being of middle-aged participants [41].

Guidelines for PA have acknowledged the health benefits of moderate-to-vigorous PA (MVPA), particularly for adults with MetS and body mass management [5, 42]. The risk to develop Mets in middle-aged adults is lowered by 64% when engaged in MVPA for ≥ 30 minutes/day [42]. In this study, a 60-minute FFH produced ~42 minutes of MVPA that corresponds to 70% of the total duration of an FFH. This exceeds the recommended duration of MVPA by approximately 15 minutes [42], suggesting a potential beneficial effect for adults with MetS (figure 7C).

This is the first attempt to determine the total energy cost induced by FFH in middle-aged males with MetS. Prior studies that have estimated the energy cost during exercise have used a novel algorithm based on accelerometer, exercise type, and demographic data [16], or a linear regression analysis based on exercise ${\dot{\text{V}}\text{O}}_{2}$ and HR [22], or bracelets and new software from footwear-based devices [36]. However, the estimation of TEE includes ${\dot{\text{V}}\text{O}}_{2}$ consumption during exercise, La concentration, and EPOC [30], due to the aerobic-anaerobic nature of football [7]. Using this methodology, TEE was estimated at 524 kcal. The relative contribution of MEP, AS, and EPOC to TEE was estimated to be 90%, 2%, and 8% respectively, revealing a higher contribution of MEP relative to the other two sources. These results suggest that FFH mainly relies on mitochondrial energy sources, probably because when performed by middle-aged individuals with MetS, its intensity is lower, as indicated by the GPS data.

According to the Physical Activity Guidelines for Americans and the ASCM guidelines, an energy expenditure of approximately 1000 kcal/week is associated with a marked reduction of cardiometabolic risk [8]. A single FFH produces a caloric deficit of ~500 kcal, or 52.4% of the total weekly energy expenditure recommended by current guidelines. Hence, based on estimation and excluding the possibility of fatigue, two or three FFH practices/week would induce a total energy deficit that would surpass the target energy expenditure of 1000 kcal/week by 105% or 157%, respectively (figure 7D). According to current recommendations [32], a PA protocol that induces an energy expenditure between 8 and 12 kcal/kg/week is more effective for the management of MetS (i.e. reduction of SBP, DBP, waist circumference, triglycerides and fasting glucose by 2.3%, 1.3%, 2.3%, 3.9%, and 1.8%, respectively) compared to a 4 kcal/kg/week protocol that is associated with a rise in SBP and DBP by 0.8% and 1.2% and a reduction of waist circumference, triglycerides, and fasting glucose by 1.0, 5.5 and 0.9%, respectively) [4]. Here, one FFH approached an energy expenditure of 6 kcal/kg suggesting that the addition of a second session of FFH would induce a caloric expenditure of ~12 kcal/kg/week which would be effective in MetS management. This statement is further supported by a meta-analysis showing positive effects on most MetS components after 12–16 weeks of training (2 weekly sessions) [11]. Most published FFH’s are performed twice a week [13], a frequency that meets most guidelines for PA for adults with MetS. Additionally, A recent study found that 8 weeks of recreational football or basketball significantly improved aerobic capacity and muscular strength in sedentary overweight and obese men and women. These results support the use of recreational team-based formats, as effective tools for improving physical fitness and managing metabolic risk [43].

Enjoyment, variety and sociality are the main factors for long-duration participation in physical activity programs, particularly among inactive individuals. Specifically, it has been previously demonstrated that high-intensity interval neuromuscular training improves psychological indicators in inactive obese women, highlighting the importance of enjoyable exercise formats [29, 44]. Similarly, recreational football could promote sociality and motivation, with participants reporting a lower perception of exertion and a higher enjoyment compared to the traditional exercise types [45].

This research advances knowledge compared to previous studies, using direct physiological and activity measurement to quantify TEE, including the anaerobic and EPOC components, for a clinical population (middle-aged men with metabolic syndrome). Furthermore, it determines the results according to international PA guidelines for MetS management, including practical implications for the use of football training as an effective, engaging, and time-efficient exercise intervention to reduce cardiometabolic risk. Also, this study represents significant progress in understanding the energy cost and health relevance of football training for populations with metabolic risk factors, thus supporting exercise prescriptions customized to MetS patients.

Limitations of this study include the lack of assessment of gender differences in the response to FFH. Furthermore, BL concentrations were assessed in this study before and after the FFH but not during training. Given that football is a high-intensity intermittent activity with a high frequency of activity changes and a varied movement pattern, our measurements might have underestimated the contribution of non-mitochondrial glycolysis to total energy expenditure. Also, another limitation of this study is that the results in Figure 7 are based on estimates from one FFH and not direct measurements from multiple practices. The execution of FFH two or three times per week could lead to fatigue, reducing the intensity and TEE in the next practices.

## CONCLUSIONS

In conclusion, this study is the first to examine the TEE during an FFH in middle-aged males with MetS. The data analysis suggests that FFH can be a highly effective form of PA, with the potential to significantly contribute to overall energy expenditure. Furthermore, according to the metabolic load, physiological responses, and field activity, a 60-min FFH session is characterized as a moderate to vigorous-intensity exercise and is associated with a TEE of > 500 kcal. These findings suggest that FFH is a reliable and engaging training method that, when performed at least twice per week, can meet international recommendations for PA to improve cardiometabolic health in adults with MetS. Future investigations should investigate the energy expenditure and PA associated with different training formats in both males and females with MetS.
